# Association between cooling temperature and outcomes of patients with heat stroke

**DOI:** 10.1007/s11739-023-03291-y

**Published:** 2023-05-03

**Authors:** Lan Chen, Shuying Xu, Xiaoling Yang, Junlu Zhao, Yuping Zhang, Xiuqin Feng

**Affiliations:** 1 Nursing Department, The Second Affiliated Hospital of Zhejiang University School of Medicine, 88 Jiefang Road, Shangcheng District, Hangzhou, 310009 Zhejiang China; 2 Emergency Department, Dongyang People’s Hospital, Dongyang, Zhejiang Province China; 3 Emergency Department, Lanxi People’s Hospital, Lanxi, Zhejiang Province China; 4 Emergency Department, Affiliated Jinhua Hospital, Zhejiang University School of Medicine, Jinhua Municipal Central Hospital, Jinhua, Zhejiang Province China

**Keywords:** Body temperature, Cooling, Hyperthermia, Hypothermia, Hospital mortality, Heat stroke

## Abstract

This study explored the relationship between body temperature and adverse outcomes in patients with heat stroke to identify the optimal target body temperature within the first 24 h. This retrospective, multicentre study enrolled 143 patients admitted to the emergency department and diagnosed with heat stroke. The primary outcome was the in-hospital mortality rate, while secondary outcomes included the presence and number of damaged organs and neurological sequelae at discharge. A body temperature curve was built using a generalized additive mixed model, and the association between body temperatures and outcomes was established by logistic regression. The threshold and saturation effects were used to explore the targeted body temperature management. Cases were divided into the surviving and non-surviving groups. The cooling rate within the first 2 h was significantly higher in the survival group than the non-survival group (β: 0.47; 95% confidence interval [CI]: 0.09–0.84; *P* = 0.014), while the non-survival group exhibited a lower body temperature within 24 h (β: − 0.06; 95% CI: − 0.08 to − 0.03; *P* ≤ 0.001). Body temperature after 2 h (odds ratio [OR]: 2.27; 95% CI: 1.14–4.50; *P* = 0.019) and lowest temperature within 24 h (OR: 0.18; 95% CI: 0.06–0.55; *P* = 0.003) were significantly related to in-hospital mortality rate. When the body temperature at 0.5 h was 38.5–40.0 °C, the number of damaged organs was at its lowest. In patients with heat stroke, both hyperthermia and hypothermia were associated with adverse outcomes. Hence, an accurate body temperature management is required during the early stages of care.

**Supplementary Information:**

The online version contains supplementary material available at 10.1007/s11739-023-03291-y.

## Introduction

Body temperature (BT) is a critical variable in health and disease, with the optimal temperature for normal cellular function falling within one to two degrees of 37 °C [1]. Environmental conditions that overwhelm our bodies’ thermoregulatory capacity can lead to severe reductions or elevations in BT [1], carrying substantial risk of neurological dysfunctions and posing a threat to life [1, 2]. Heat stroke (HS) is the most dangerous heat-related illness associated with weather conditions, especially extremely high temperature and humidity [3–5]. Hyperthermia causes progressive cellular dysfunction, resulting in multi-organ failure and death [6], the severity of the injury being dependent on the highest temperature experienced and exposure duration [7–9]. Hence, it is imperative to use immediate cooling and organ support management of to prevent irreversible damage and death in patients with HS [10, 11].

Lowering the core BT blocks the cellular damage [12], while delayed BT cooling or slower BT recovery can lead to neurological damage and significantly higher fatality rates [12, 13]. However, the most optimal cooling target values for HS remain controversial. Laitano et al. [12] suggested that lowering the BT to less than 40.5 °C within the first 30 min after the HS collapse is optimal to prevent tissue injuries, while other studies suggested a value of 40.0 °C to prevent death effectively [14, 15]. However, these studies focused on the on-site emergency treatments for exertional HS (EHS). Regarding hospitalised patients, Liu et al. [16] found that, if BT did not effectively cool (< 38.0 °C) within 4.88 h, patients had a higher risk of in-hospital death. In fact, Yokobori et al. [17] considered a temperature of 37 °C within the first 24 h as optimal values, arguing that these parameters significantly reduce the Sequential Organ Failure Assessment (SOFA) scores. The HS expert consensus in China recommends that efforts should be made to decrease the core temperature to ≤ 39 °C within 10–40 min and to ≤ 38.5 °C within 2 h [18], since setting different cooling targets for different time points increases the effectiveness of the medical staff. However, the relationship between such targeted temperature values and HS prognosis remains unclear.

Aggressive cooling can cause a rapid decrease in the BT to < 37 °C in patients with HS [19]. However, a decline close to hypothermia can lead to abnormal coagulation, acidosis, hyperkalaemia, and elevated serum creatinine [2]. Therefore, we must prevent hypothermia during the active cooling step [20, 21], although the precise cut-off values remain controversial. The proposed range was from 37 to 39 °C [12, 19–22], but most values were not based on scientific data [12], and only a few studies have explored the incidence of hypothermia and the impact of a low BT on the HS prognosis.

With climate change, HS-induced deaths are increasing each year [18], and a more precise BT management is required to improve the outcomes [17, 18]. To improve the HS management, we explored the relationship between the BT and adverse outcomes, aiming to identify the optimal target BT in the first 24 h.

## Methods

### Participants

This retrospective, multicentre study was performed in four tertiary-care hospitals in Zhejiang Province, China, from January 1, 2021 to September 30, 2022. This study was approved by the Ethics Committee of the Second Affiliated Hospital of Zhejiang University School of Medicine (approval 2022-0913). The requirement for informed consent was waived because the data were anonymous. The STROBE guidelines for cohort studies were applied.

Only the patients admitted to the emergency departments diagnosed with HS based on the expert consensus [23] were enrolled in this study. The diagnostic criteria of HS that was agreed upon had two aspects, i.e., “medical history information” and “clinical presentation.” The medical history information criterion includes high-intensity exercise and exposure to high temperature and high humidity environments, while the clinical presentation criterion includes core temperatures above 40 °C, central nervous system functional impairment, multiple organ (≥ 2) functional impairment, and severe coagulopathy or disseminated intravascular coagulation (DIC). HS was considered if the patient met any medical history information and clinical presentation criteria. The HS consensus in China no longer considers core temperature as a necessary diagnostic component to minimize any treatment delays. Since this study aimed to explore the relationship between the BT and adverse outcomes, those patients transferred from another hospital and BT < 39.5 °C, those discharged within 4 h, and with unknown subsequent BT or outcome were excluded from this study. Patients were also excluded if they required immediate cardiopulmonary resuscitation or had other serious diseases, such as massive cerebral haemorrhage or severe trauma.

### Data collection

Information on the demographics, signs, symptoms, onset time, underlying comorbidities, cooling method, HS type, and outcomes of patients were retrieved from the electronic medical records. The maximum ambient temperature and relative humidity values on the day of treatment were recorded, and the humidex value was calculated [24].

BT was measured using infrared ear thermometers, which introduced some irregularity in recording intervals. BT was recorded during the first 24 h after admission, and initial BT was documented at admission. BT at 0.5 h refers to a reading obtained 25–35 min after admission. BT at 2 h refers to a reading taken 100–140 min after admission. The highest and lowest temperatures within 24 h were obtained from the medical record. The cooling rate at 0.5 h was calculated in reference to initial temperature and BT at 0.5 h.

### Outcomes

The primary outcome of this study was in-hospital mortality rate. Secondary outcomes included the presence and number of damaged organs and neurologic sequelae at discharge. Organ damage was defined as any occurrence of acute kidney, liver, or heart failure, respiratory failure, disseminated intravascular coagulation, rhabdomyolysis, or neurologic sequelae at discharge, as these are the main categories of HS-related organ damage [6]. Acute kidney injury (AKI) was included in the acute renal failure category, since it is a significant factor associated with the prognosis of HS [25]. It was defined as an increase in the serum creatinine levels by 50% within 7 days, with 0.3 mg/dl (26.5 μmol/l) within 2 days, or oliguria [26]. An acute heart failure diagnosis was determined when the patient presented acute signs and symptoms of heart failure, including dyspnea, orthopnea, lower limb swelling, elevated jugular venous pressure, and pulmonary congestion [27]. Acute respiratory failure was based on an oxygenation index less than 200 and the need for mechanical respiratory support [28]. The number of damaged organs was defined as the sum of the above indicators. Neurologic sequelae were defined as the deterioration of the nervous system function compared to the patient’s expected baseline, including cognitive impairment, language disorder, and ataxia.

### Statistical analysis

Empower 4.0 software was used for analysis (www.empowerstats.com, X&Y Solutions; Boston, MA) and R 3.6.3 (http://www.R-project.org). Statistical significance was defined as a two-tailed *P*-value < 0.05. Baseline characteristics and patient outcomes were summarised using descriptive statistics. Categorical variables were reported as numbers (percentages) and were analysed using the chi-square or Fisher’s exact tests, as appropriate. Normally distributed continuous variables were compared using the Student’s t-test and were expressed as the mean with standard deviation, while non-normally distributed data were compared using the Mann–Whitney U test and were expressed as the median with interquartile range.

To explore the association between time-specific BT and outcomes, we first used a generalised additive mixed model (GAMM) to describe the BT curve within 24 h, followed by logistic regression to identify the specific association between the temperature and each outcome. Finally, the threshold and saturation effects analysis explored the target temperature at each time point.

The GAMM was used to analyse the longitudinal BT values within the first 2 and 24 h after the admission and the differences in the BT change between the survival and non-survival groups. GAMM is ideal for analysing longitudinal data due to its flexibility in modelling time effects, appropriate handling of the missing data, and accommodation of the unbalanced and unevenly spaced time observations [29].

The adverse outcomes were analysed using logistic regression, with the results presented as odds ratios (ORs) with 95% confidence intervals (CIs), a multivariate sensitivity analysis was performed for all outcomes, and the BT readings at 0.5, 2, and 24 h were analysed as continuous variables in all regressions. Because BT changed quickly in the first half hour, we divided the temperatures at 0.5 h into three groups for analysis (≤ 39 °C, 39.1–40 °C, > 40 °C). The BT readings at 2 and 24 h were divided into two groups, (≤ 38.5 °C, > 38.5 °C) and (≤ 36 °C, > 36 °C), respectively. In the adjusted models, covariates were retained if they changed the estimated effect of the BT on an outcome by more than 10% or if they were independently associated with an outcome. Initial BT was also included as a covariate because subsequent BT values were highly dependent on the initial temperatures. Nevertheless, we did not include any laboratory results as confounders since they were manifestations of the organ dysfunctions. Supplementary Tables S1–S4 show the associations between each confounder and the outcomes of interest and changes in the estimated effect.

Finally, we selected the number of damaged organs as the dependent variable to identify the optimal target BT at 0.5, 2, and 24 h based on our assumption that the best treatment goal is the reduction of the organ dysfunction risks, not only death. To achieve this, we first used a smoothed curve fitting to examine whether the BT changes were partitioned into intervals. Next, we applied the segmented regression analysis, which uses a separate line segment to fit each interval, and a log-likelihood ratio test by comparing the single line non-segmented and segmented regression models to determine the threshold.

## Results

### Patient characteristics

**Fig. 1:**
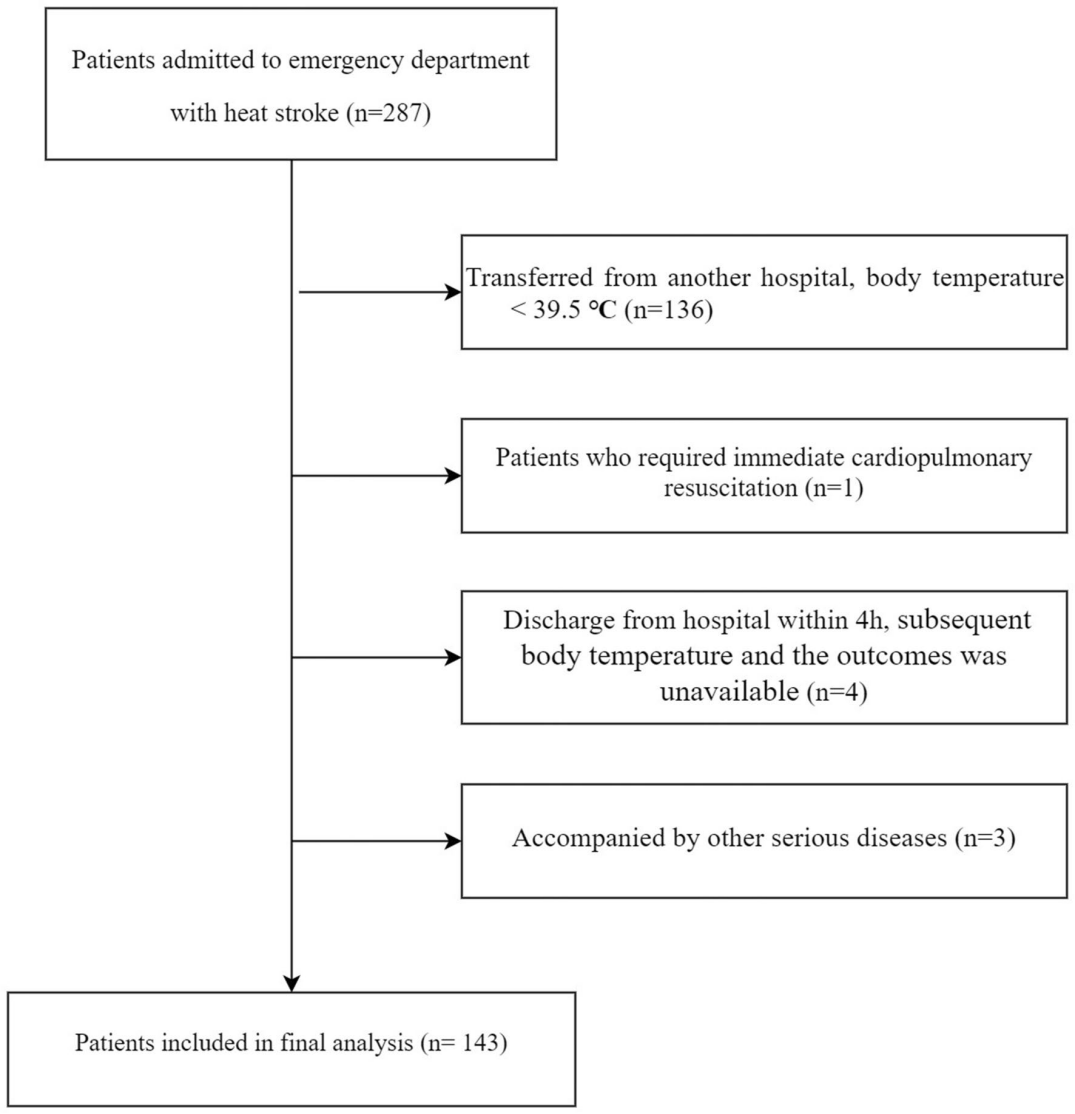
Flow chart. Initially, 287 patients were recruited, but only 143 patients remained after applying the exclusion criteria

The cohort included 143 patients (Fig. 1, Table 1) with a median age of 66.83 ± 15.03 years, of which 56 (39.16%) were women. Overall, 55 (38.46%) patients exhibited classic HS (CHS) and 88 (61.54%) EHS. The mean ambient temperature and humidex values were 38.66 ± 3.44 °C and 56.09 ± 6.94, respectively. Hypertension (22.38%) was the most common comorbidity, followed by mental disorders (13.29%) and stroke (8.39%). Loss of consciousness occurred in 119 (83.22%) patients, and the Glasgow Coma Scale score at admission was 8.70 ± 4.14.

**Table 1:** Patient characteristics

Variables	All (N = 143)	In-hospital mortality		*P*-value
		Survivors (n = 130)	Non-survivors (n = 13)	
Baseline characteristics				
Age (years)	66.83 ± 15.03	66.36 ± 15.10	71.46 ± 14.04	0.25
Female, n (%)	56 (39.16)	48 (36.92)	8 (61.54)	0.08
Heatstroke type, n (%)				
Classic	55 (38.46)	50 (38.46)	5 (38.46)	1.00
Exertional	88 (61.54)	80 (61.54)	8 (61.54)	
Ambient temperature, ( °C)	38.66 ± 3.44	38.62 ± 3.57	39.00 ± 1.58	0.71
Relative humidity (%)	60.16 ± 11.90	60.36 ± 11.85	58.23 ± 12.72	0.444
Humidex	56.09 ± 6.94	56.11 ± 7.20	55.86 ± 3.63	0.630
Underlying illness, n (%)				
Diabetes	7 (4.90)	5 (3.85)	2 (15.38)	0.12
Stroke	12 (8.39)	11 (8.46)	1 (7.69)	0.92
Mental disorder	19 (13.29)	16 (12.31)	3 (23.08)	0.28
Coronary heart disease	9 (6.29)	8 (6.15)	1 (7.69)	0.59
Hypertension	32 (22.38)	29 (22.31)	3 (23.08)	0.95
Kidney failure	1 (0.70)	0 (0.00%)	1 (7.69)	0.09
Initial manifestations, n (%)				
Loss of consciousness	119 (83.22)	107 (82.31)	12 (92.31)	0.36
Convulsions	15 (10.49)	14 (10.77)	1 (7.69)	0.73
Time from onset to visit (h)	1.00 (1.00–3.00)	1.50 (1.00–3.38)	1.00 (1.00–2.00)	0.28
Monitoring parameters at admission				
Heart rate (beat/min)	130.06 ± 24.12	129.33 ± 24.36	137.31 ± 21.02	0.26
Respiratory rate (beat/min)	27.52 ± 9.19	27.66 ± 9.17	26.08 ± 9.66	0.56
Systolic pressure (mmHg)	126.85 ± 31.49	127.72 ± 29.38	118.15 ± 48.65	0.30
Diastolic pressure (mmHg)	69.34 ± 21.26	69.36 ± 19.56	69.08 ± 35.16	0.96
Pulse oxygen saturation (%)	91.66 ± 6.79	92.25 ± 6.23	85.85 ± 9.33	0.001
Glasgow Coma Scale	8.70 ± 4.14	9.09 ± 4.12	4.77 ± 1.30	< 0.001
Cooling methods, n (%)				
Evaporation	45 (31.47)	40 (30.77)	5 (38.46)	0.57
Cold packs	70 (48.95)	64 (49.23)	6 (46.15)	0.83
Cooling blankets	52 (36.36)	46 (35.38)	6 (46.15)	0.44
Cold fluids infused	140 (97.90)	127 (97.69)	13 (100.00)	1.00
Iced gastric/bladder lavage	4 (2.80)	4 (3.08)	0 (0.00)	1.00
Body temperature (°C)				
Initial temperature	40.74 ± 0.76	40.73 ± 0.75	40.88 ± 0.81	0.51
Temperature (0.5 h)	39.34 ± 0.94	39.32 ± 0.89	39.52 ± 1.35	0.47
Temperature (2 h)	37.81 ± 0.96	37.73 ± 0.85	38.55 ± 1.50	0.003
Highest temperature	40.78 ± 0.77	40.76 ± 0.75	41.04 ± 0.86	0.21
Lowest temperature within 24 h	36.39 ± 0.70	36.44 ± 0.66	35.89 ± 0.91	0.006
Cooling rate (0.5 h) °C/min	0.04 (0.02–0.07)	0.04 (0.02–0.08)	0.04 (0.02–0.05)	0.38
Temperature > 39 °C (0.5 h), n (%)	85 (62.50)	76 (61.79)	9 (69.23)	0.60
Temperature > 38.5 °C (2 h), n (%)	29 (21.32)	23 (18.70)	6 (46.15)	0.02
Outcomes				
Consciousness recovery time (d)	0.50 (0.00–3.50)	0.50 (0.00–2.00)	4.00 (3.00–6.00)	< 0.001
Sepsis, n (%)	19 (13.29)	13 (10.00)	6 (46.15)	< 0.001
Acute kidney failure, n (%)	53 (37.06)	42 (32.31)	11 (84.62)	< 0.001
Acute liver failure, n (%)	43 (30.07)	37 (28.46)	6 (46.15)	0.19
Acute heart failure, n (%)	19 (13.29)	13 (10.00)	6 (46.15)	< 0.001
Respiratory failure, n (%)	45 (31.47)	34 (26.15)	11 (84.62)	< 0.001
Disseminated intravascular coagulation, n (%)	4 (2.80)	2 (1.54)	2 (15.38)	0.004
Rhabdomyolysis, n (%)	13 (9.09)	13 (10.00)	0 (0.00)	0.23
Neurologic sequelae at discharge, n (%)	21 (14.69)	9 (6.92)	12 (92.31)	< 0.001
Multiple organ dysfunction syndrome, n (%)	8 (5.59)	4 (3.08)	4 (30.77)	< 0.001
Organs damaged, n (%)	83 (58.04)	70 (53.85)	13 (100.00)	0.001
Number of damaged organs, n (%)	1.00 (0.00–2.00)	1.00 (0.00–2.00)	4.00 (3.00–5.00)	< 0.001

### BT management

Among cooling methods, 97.90% of the cases used fast infusion of cold fluids. Other methods included evaporation, cold packs, and cooling blankets, and 103 patients received two or more interventions. Initial temperature, BT at 0.5 h, and BT at 2 h were 40.74 ± 0.76 °C, 39.34 ± 0.94 °C, and 37.81 ± 0.96 °C, respectively. The lowest temperature within 24 h was 36.39 ± 0.70 °C. According to the consensus criteria, 85 (62.50%) patients did not reach the 30-min target temperature (≤ 39.0 °C), and 29 (21.32%) did not reach the 2-h target temperature (≤ 38.5 °C). The cooling rate during the first 30 min was 0.04 (0.02–0.07) °C/min.

### Adverse outcomes

Acute kidney failure (37.06%) was the most common complication, followed by respiratory failure (31.47%) and acute liver failure (30.07%). Additionally, 21 (14.69%) patients suffered from neurological sequelae at discharge, and 83 (58.04%) patients had one or more damaged organs. Moreover, 13 (9.1%) patients experienced in-hospital mortality, of which 5 died in the first 24 h, and the rest died because of multiple organ dysfunction syndrome (MODS). Compared to the survivors, the non-survivors had significantly lower pulse oxygen saturation (92.25% vs. 85.85%, *P* = 0.001) and Glasgow Coma Scale scores (9.09 vs. 4.77, *P* < 0.001).

### BT change within 24 h

**Fig. 2:**
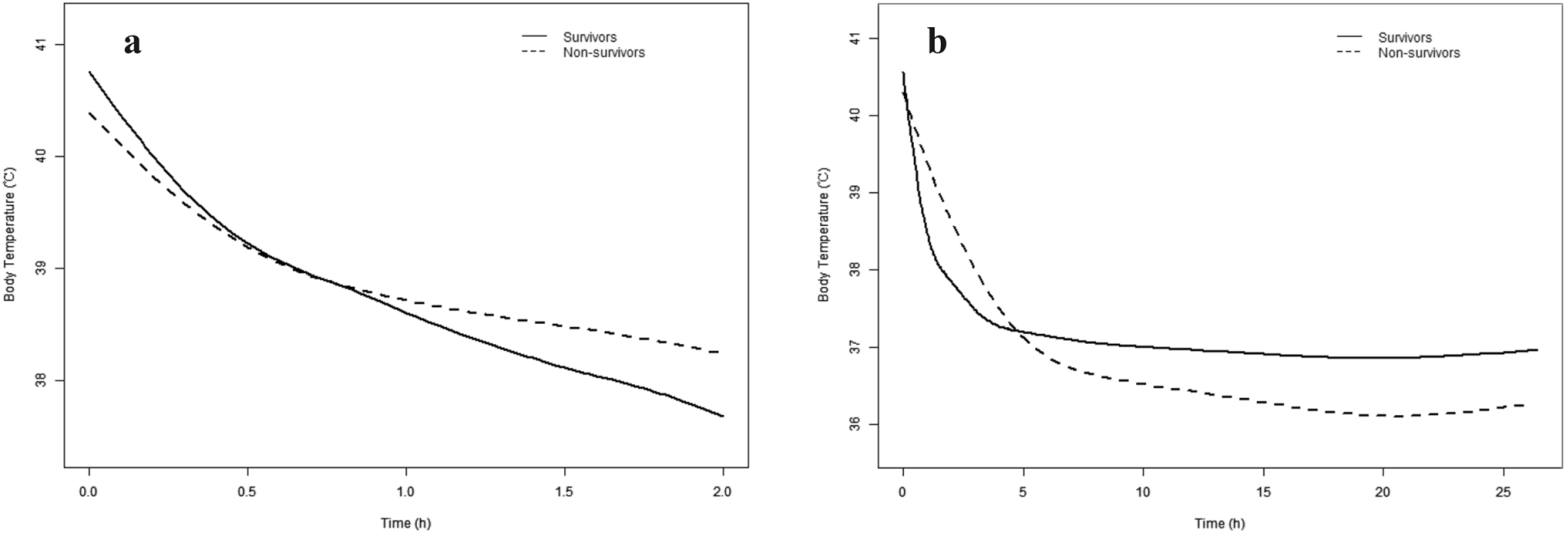
Association between body temperature within 2 h and 24 h and mortality using the generalized additive mixed model. **A** The impact of body temperature within the first 2 h was significantly affected by the mortality type (*P* for interaction = 0.01). The body temperature within the first 2 h decreased by 0.47 °C (95% confidence interval: 0.09–0.84) every hour in the survival group compared to the non-survival group. **B** The impact of body temperature within the first 24 h was significantly affected by the mortality type (*P* for interaction ≤ 0.001). The body temperature within the first 24 h decreased by 0.06 °C (95% confidence interval: − 0.08 to − 0.03) every hour in the non-survival group compared to the survival group

The GAMM showed that BTs within the first 2 and 24 h significantly decreased with time across all patients and subgroups (Table 2). However, the cooling rate within the first 2 h was significantly higher in the survival than non-survival group (β: 0.47; 95% CI: 0.84–0.09; *P* = 0.014), while the non-survival group had a lower BT within 24 h (β: − 0.06; 95% CI: − 0.08 to − 0.03; *P* ≤ 0.001) than the survival group. After the initial rapid cooling step, the BT stabilised at a higher level in the survival group, while the non-survival group cooled initially more slowly, followed by a gradual dropping to a lower level (Fig. 2A, B). This phenomenon was also seen in both the CHS and EHS subgroups (Supplementary Fig. 1A-B).

**Table 2:** Longitudinal body temperature from a linear mixed-effects regression model

Variables	Body temperature within 2 h		Body temperature within 24 h	
	β (95% CI)	*P*-value	β (95% CI)	*P*-value
Time (all patients)	39.27 (39.14 to 39.40)	< 0.001	37.94 (38.03 to 37.85)	< 0.001
Time (survival group)	− 3.11 (− 3.34 to − 2.89)	< 0.001	− 2.34 (− 2.10 to 2.58)	< 0.001
Time (non-survival group)	− 2.15 (− 2.90 to − 1.40)	< 0.001	− 3.77 (− 4.43 to 3.11)	< 0.001
Time × survival vs. non-survival (test for interaction)	0.47 (0.84 to 0.09)	0.01	− 0.06 (− 0.08 to 0.03)	< 0.001

### BT and adverse outcomes

To assess whether temperatures at different times were a risk factor for adverse outcomes, logistic regression was performed. In the univariate analysis, BT at 0.5 h was associated with the number of damaged organs (Table 3). The temperature at 2 h and the lowest temperature within 24 h were associated with all primary and secondary outcomes.

**Table 3:** Logistic regression of body temperature for outcomes in patients with heat stroke

Body temperature	Outcomes							
	In-hospital mortality		Organs damaged		Number of damaged organs		Neurological sequelae at discharge	
	Univariate	Adjusted model	Univariate	Adjusted model	Univariate	Adjusted model	Univariate	Adjusted model
	OR (95% CI) P	OR (95% CI) P	OR (95% CI) P	OR (95% CI) P	β (95% CI) P	β (95% CI) P	OR (95% CI) P	OR (95% CI) P
Temperature (0.5 h)								
Per 1 °C increase	1.26 (0.68–2.34) 0.468	0.95 (0.41–2.18) 0.903^a^	1.29 (089–1.88) 0.176	1.24 (0.77–2.00) 0.382^d^	0.27 (0.19–0.36) < 0.001	0.15 (0.05–0.26) 0.005^g^	1.38 (0.28–2.30) 0.221	1.08 (0.57–2.06) 0.816^j^
≤ 39 °C	1.0	1.0	1.0	1.0	0	0	1.0	1.0
39.1–40 °C	0.92 (0.22–3.90) 0.912	0.73 (0.12–4.39) 0.730	1.44 (0.67–3.11) 0.351	1.21 (0.49–2.98) 0.681	0.28 (0.08–0.47) 0.005	0.03 (− 0.19–0.26) 0.786	0.92 (0.30–2.82) 0.879	0.56 (0.13–2.36) 0.433
> 40 °C	2.35 (0.58–9.54) 0.232	1.08 (0.14–8.51) 0.941	2.64 (0.99–7.03) 0.051	2.58 (0.73–9.14) 0.142	0.62 (0.40–0.84) < 0.001	0.39 (0.11–0.67) 0.006	1.91 (0.60–6.12) 0.274	1.02 (0.19–5.51) 0.978
Temperature (2 h)								
Per 1 °C increase	2.25 (1.25–4.06) 0.007	2.27 (1.14–4.50) 0.019^b^	1.68 (1.12–2.52) 0.012	1.67 (1.02–2.73) 0.040^e^	0.44 (0.35–0.52) < 0.001	0.33 (0.24–0.43) < 0.001^h^	2.04 (1.23–3.36) 0.006	2.10 (1.11–4.00) 0.023^k^
≤ 38.5 °C	1.0	1.0	1.0	1.0	0	0	1.0	1.0
> 38.5 °C	3.73 (1.14–12.14) 0.029	3.18 (0.83–12.24) 0.093	2.76 (1.09–7.00) 0.033	3.06 (1.02–9.22) 0.047	0.76 (0.57–0.96) < 0.001	0.69 (0.47–0.90) < 0.001	3.56 (1.32–9.58) 0.012	5.32 (1.27–22.25) 0.022
Lowest temperature within 24 h								
Per 1 °C increase	0.34 (0.15–0.76) 0.009	0.18 (0.06–0.55) 0.003^c^	0.41 (0.24–0.71) 0.002	0.49 (0.24–0.98) 0.044^f^	− 0.70 (− 0.82 to − 0.59) < 0.001	− 0.56 (− 0.71 to − 0.41) < 0.001^i^	0.34 (0.17–0.67) 0.002	0.31 (0.12–0.78) 0.013^l^
≤ 36 °C	1.0	1.0	1.0	1.0	0	0	1.0	1.0
> 36 °C	0.41 (0.13–1.32) 0.135	0.32 (0.09–1.22) 0.096	0.30 (0.13–0.68) 0.004	0.38 (0.13–1.08) 0.069	− 0.72 (− 0.90 to − 0.55) < 0.001	− 0.4 (− 0.60 to − 0.18) < 0.001	0.28 (0.11–0.73) 0.009	0.37 (0.11–1.26) 0.112

^a^Model adjusted for initial temperature, sex, age, HR, SBP, SPO_2_, GCS, time from onset to admission, kidney failure, diabetes, and mental disorders

^b^Model adjusted for initial temperature, sex, age, SPO_2_, GCS, stroke type, diabetes, and mental disorders

^c^Model adjusted for initial temperature, sex, HR, SPO_2_, GCS, kidney failure, diabetes, and mental disorders

^d^Model adjusted for initial temperature, HR, SBP, SPO_2_, GCS, and time from onset to admission

^e^Model adjusted for initial temperature, HR, SBP, SPO_2_, GCS, and hypertension

^f^Model adjusted for initial temperature, HR, SBP, SPO_2_, GCS, and diabetes

^g^Model adjusted for initial temperature, HR, SBP, SPO_2_, GCS, diabetes, and mental disorders

^h^Model adjusted for initial temperature, HR, SBP, SPO_2_, GCS, and mental disorders

^i^Model adjusted for initial temperature, HR, SBP, SPO_2_, GCS, and mental disorders

^j^Model adjusted for initial temperature, age, HR, SBP, SPO_2_, GCS, diabetes, stroke, mental disorders, coronary heart disease, and hypertension

^k^Model adjusted for initial temperature, age, HR, SBP, SPO_2_, GCS, diabetes, and mental disorders

^l^Model adjusted for initial temperature, HR, SBP, SPO_2_, GCS, kidney failure, diabetes, and mental disorders

*CI* confidence interval, *HR* heart rate, *SBP* systolic pressure, *SPO*_*2*_ pulse oxygen saturation, *GCS* Glasgow Coma Scale

Multivariable logistic regression models were adjusted based on the screening criteria. The multivariate analysis indicated that the number of damaged organs increased 15% for every 1 °C-increase in BT at 0.5 h (95% CI: 0.05–0.26; *P* < 0.001). Compared to those whose BT at 0.5 h was ≤ 39 °C, patients whose temperature was > 40 °C maintained a higher risk of organ damage (β: 0.39; 95% CI: 0.11–0.67; *P* = 0.006). Moreover, BT at 2 h was related to in-hospital mortality (OR: 2.27; 95% CI: 1.14–4.50; *P* = 0.02), organ damage (OR: 1.67; 95% CI: 1.02–2.73; *P* = 0.04), number of damaged organs (β: 0.33; 95% CI: 0.24–0.43; *P* < 0.001), and neurological sequelae (OR: 2.10; 95% CI: 1.11–4.00; *P* = 0.02). The association between the lowest temperature within 24 h and adverse outcomes was also significant. As the lowest temperature within 24 h increased, the risk of in-hospital mortality (OR: 0.18; 95% CI: 0.06–0.55; *P* = 0.003), risk of damaged organs (OR: 0.49; 95% CI: 0.24–0.98; *P* = 0.04), number of damaged organs (β: − 0.56; 95% CI: − 0.71 to − 0.41; *P* < 0.001), and neurologic sequelae (OR: 0.31; 95% CI: 0.12–0.78; *P* = 0.01) decreased.

### Optimal BT over time

**Fig. 3:**
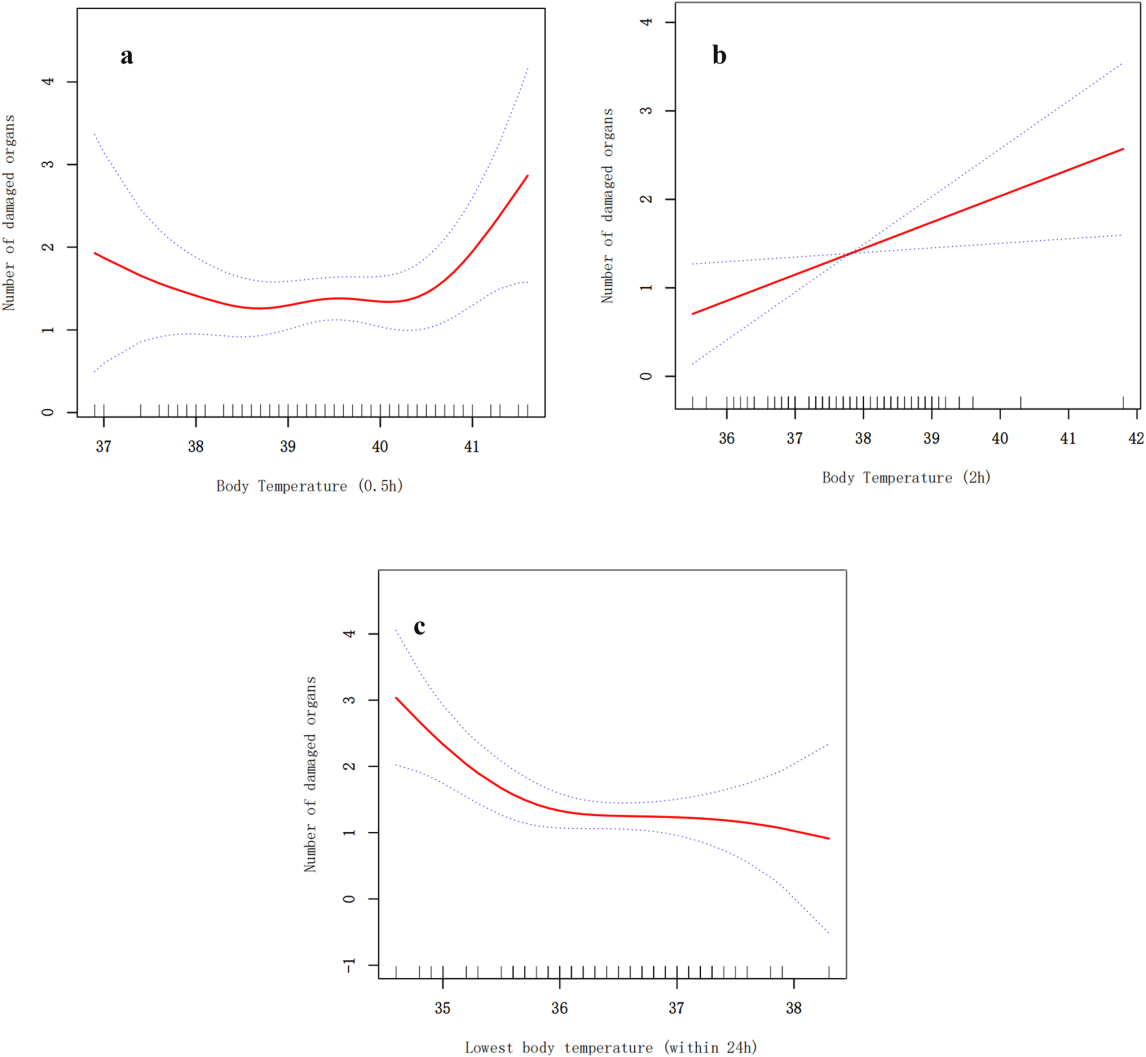
Smooth curve of body temperature and the number of damaged organs after adjustment. Dashed lines indicate 95% confidence intervals. **A** The association between body temperature at 0.5 h and number of damaged organs. The relationship was significantly different when the body temperature was < 38.5 °C, 38.5–40.0 °C, or > 40.0 °C. **B** The association between body temperature at 2 h and number of damaged organs was nearly a straight line. **C** The association between lowest body temperature and number of damaged organs was different when the body temperature was above or below 36 °C

We performed a smoothed function analysis to explore the potential nonlinear association between the BT and the number of damaged organs. After adjustment, the model showed a U-shaped relationship between the BT at 0.5 h and the number of damaged organs; the threshold effects being shown by the segmented regression (Fig. 3A, Supplementary Table S5). When BT at 0.5 h exceeded 40.0 °C, the number of damaged organs increased rapidly upon further BT increases (β: 1.42; 95% CI: 0.29–2.56; *P* = 0.02), while when they between 38.5 and 40.0 °C, the number of damaged organs remained stable (β: 0.27; 95% CI: − 3.1–0.86; *P* = 0.36). However, when the BT at 0.5 h was < 38.5 °C, the number of damaged organs increased with any additional decrease in the BT values (β: − 0.57; 95% CI: − 1.54–0.40; *P* = 0.25), the difference being statistically significant among the three segments (*P* = 0.007).

As shown in Fig. 3B, the association between the BT at 2 h and the number of damaged organs was almost linear, and the threshold effects could not be determined. Regarding the lowest BT within 24 h, the risk of organ damage decreased when the lowest temperature increased (Fig. 3C), with an inflection point at 36 °C. The β (95% CI) was 0.02 (− 0.47–0.51) on the right side of the inflection point and − 1.25 (− 2.01 to − 0.49) on the left side. The intergroup difference was statistically significant (*P* = 0.02).

## Discussion

In this retrospective, multicentre study of patients with HS, the cooling rates of the first 2 and 24 h were significantly different between the survivors and non-survivors. The BT at 2 h and the lowest temperature within 24 h were related to the in-hospital mortality, presence of organ damage, number of damaged organs, and neurological sequelae at discharge. In comparison, BT at 0.5 h was related to the number of damaged organs, but the lowest temperature within 24 h was the most important factor affecting the outcome. The optimal BT at 0.5 h was 38.5–40.0 °C, and the ideal lowest BT was > 36 °C.

Human health is inextricably linked to climate change, and its most direct effects are heat stress and other related disorders [4]. As the frequency, severity, and duration of extreme heat events increase [30], the number of heat-related deaths and illnesses are also increasing every year, which is becoming a major health challenge [4, 18]. HS is the most dangerous heat-related illness, and mainly occurs in extreme heat and humid weather conditions [3–5]. Humidex, which incorporates air temperature and humidity, is a well-known heat index that is used to quantify heat exposure [24], and its increase is correlated with the risks of heat-related diseases. Therefore, when humidex is above 40, exertion should be avoided, and when it is above 45, attention should be paid to the risk of HS [31]. In our study, the mean humidex values were 56.09, and 138 (96.50%) patients collapsed during the day it was above 45. Therefore, to reduce the impact of climate change, it is necessary to design early warnings and response management systems based on humidity and heat index values.

Although the BT criterion for diagnosis remains controversial, a higher core temperature is a basic element of HS [23, 32]. Consistent with our results, better temperature management may help prevent organ failure [17]. Studies have shown that a high BT is independently associated with mortality [33], however, we did not explore the association between the initial BT and the HS prognosis since we only focused on this relationship during the active cooling step. Instead, the initial BT was adjusted as a confounder to accurately explore the correlation between the cooling and prognosis. Those patients with an initial temperature < 39.5 °C were also excluded to better observe the process of temperature change process.

In addition to the in-hospital mortality, we also evaluated the presence of organ damage and number of damaged organs. The results showed that the BT at 0.5 h was associated only with the organ damage, not death, further justifying their use. Moreover, the central nervous system impairment is one of the most common HS complications [10], and some patients sustain permanent neurological damage, which affected their ability to perform daily activities and even leads to death [5, 17, 34]. Therefore, neurological sequelae at discharge were also considered.

Although many scholars have proposed cut-off points for cooling termination and argued that hypothermia should be prevented [12, 19–22], few studies have focused on the incidence of hypothermia after the cooling step and its relationship to the HS prognosis. Some researchers believe that hypothermia occurs only when BT falls below 35 °C [12], while others argue that by cooling the patients with HS to a hypothermic level might provide protection against the risk of organ damage, similarly to its usefulness in other conditions [19]. However, this study showed that a below-normal BT is significantly associated with adverse outcomes, which is consistent with previous experiments that identified hypothermia as a mortality risk factor in rodents [35, 36]. This effect may be related to the unique pathological process of HS. Coagulopathy, for example, is a common complication of severe HS [19] that is aggravated by low temperature, and the disseminated intravascular coagulation (DIC) is an independent prognostic factor for the in-hospital mortality in the patients with HS [32]. Interestingly, such patients often display hypothermia as a BT response [19], although the precise mechanism remains unclear.

BT at 0.5 h showed a U-shaped relationship with the outcome, consistent with the previous research highlighting the importance of decreasing the core temperature to < 40 °C within 30 min. Simultaneously, maintaining the cooling target at 0.5 h above 38.5 °C can prevent hypothermia after rapid cooling. However, the relationship between BT at 2 h and the outcome was almost linear, which made the determination of the threshold impossible. As the number of damaged organs decreased with decreasing BT, we speculate that BT can be further controlled at levels slightly above normal at 0.5 h. At the same time, the lowest BT within 24 h should be maintained above 36 °C to improve outcomes.

In this study, we excluded the patients with temperature at admission below 39.5 °C, which does not allow for the evaluation of the impact of the on-site or pre-hospital cooling on patient outcomes. In this study, the time from the onset-visit-admission between the survivors and non-survivors was almost similar, while the early cooling rate was significantly different, implying that the early rapid cooling in the hospital could improve the prognosis of the patients with HS. Therefore, we speculate that the on-site and pre-hospital cooling could significantly shorten the duration of overheating and offer a greater benefit for patients with HS. Studies have shown that field cooling, such as cold water immersion and cold showers, can rapidly reduce the core BT and greatly reduce EHS mortality [14, 37, 38]; however, whether field cooling could provide the same benefits to CHS is still unknown. As presented in the Supplementary Fig. 1A, it can be argued that the on-site cooling is equally important for patients with CHS. However, such analysis was not included in the aims of the present study.

To our knowledge, this is the first study to explore the relationship between the changes in BT within the first 24 h and the HS prognosis, and by setting precise temperature-per-timepoint goals, we provide a scientific basis for the HS management and benchmarks for subsequent research. Despite its strengths, this study has several limitations. First, since it was a multicentre study, subtle differences in the treatment strategies across institutions may have occurred. However, all hospitals were tertiary care centres in the same area, with uniform diagnosis and treatment standards. Second, because this was a retrospective study, we could not evaluate the causality between the BT and outcomes. Third, the BT was measured using infrared ear thermometers, and the results may differ slightly from the core temperatures measurements. However, ear thermometers have shown good accuracy so far and are widely used in clinical practice because of their convenience and non-invasiveness [39, 40]. Therefore, the results of this study are instructive for the current clinical practice. Fourth, the participants were diagnosed with HS in accordance with the expert consensus panel in China [18], for which core temperature is no longer a necessary criterion. A total of 14 participants had an initial BT < 40 °C, among whom 11 lost consciousness, 6 exhibited organ impairment, and 1 died. Moreover, we did not stratify the HS cohort by subtype; however, BT management is not differentiated by the HS subtype in clinical practice, and the target BT readings recommended by the expert consensus do not differ by subtype^18^. Finally, we were unable to identify the best cooling cut-off values since the data reflecting such specific details were missing.

## Conclusions

In our cohort, 13 (9.1%) patients experienced in-hospital mortality. Apart from five patients who died within 24 h of admission, the rest of the non-survivors died due to multiple organ dysfunction syndrome (MODS). Among the patients hospitalised with HS, the BT values at 0.5 and 2 h, as well as the lowest temperature within the first 24 h were associated with adverse outcomes. Therefore, a more precise BT management is required during the early treatment stages of HS.

## Supplementary Information

Below is the link to the electronic supplementary material.

Supplementary file1 Supplementary Fig. 1. Association between body temperature within the first 24 h and mortality using the generalized additive mixed model in different types of HS. A: CHS, B: EHS. After the initial rapid cooling, BT stabilised at a higher level in the survival group, while the non-survival group cooled more slowly and then gradually dropped to a lower temperature level. (PNG 5 KB)

Supplementary file2 (PNG 5 KB)

Supplementary file3 (DOCX 31 KB)

## Data Availability

The datasets used in this study are available from the corresponding author upon reasonable request.
