# Supplementary figures and images for: Association between cooling temperature and outcomes of patients with heat stroke
Source: Intern Emerg Med. 2023 May 3;18(6):1831–42. doi: 10.1007/s11739-023-03291-y (PMC10504196; doi:10.1007/s11739-023-03291-y)

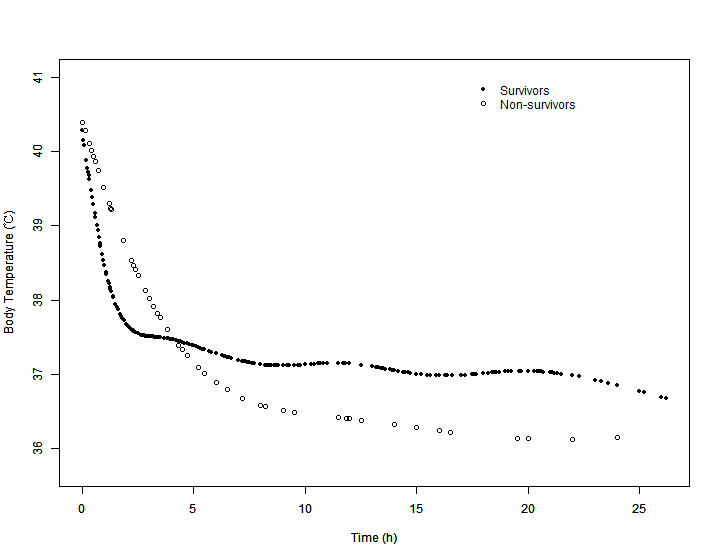

Supplement: Supplementary file 1 — Supplementary file1 Supplementary Fig. 1. Association between body temperature within the first 24 h and mortality using the generalized additive mixed model in different types of HS. A: CHS, B: EHS. After the initial rapid cooling, BT stabilised at a higher level in the survival group, while the non-survival group cooled more slowly and then gradually dropped to a lower temperature level. (PNG 5 KB) [file 11739_2023_3291_MOESM1_ESM.png]

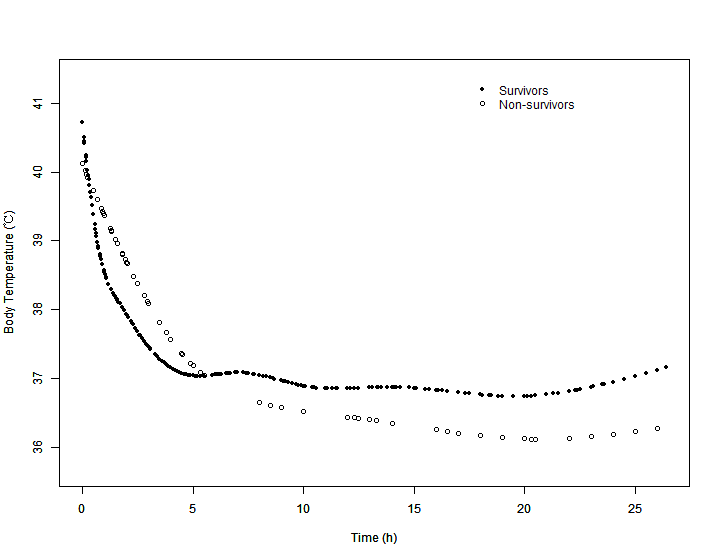

Supplement: Supplementary file 2 — Supplementary file2 (PNG 5 KB) [file 11739_2023_3291_MOESM2_ESM.png]
