# Supplementary material for: Association between cooling temperature and outcomes of patients with heat stroke
Source: Intern Emerg Med. 2023 May 3;18(6):1831–42. doi: 10.1007/s11739-023-03291-y (PMC10504196; doi:10.1007/s11739-023-03291-y)
Supplement: Supplementary file 3 — Supplementary file3 (DOCX 31 KB) [file 11739_2023_3291_MOESM3_ESM.docx]

**TABLE S1： multicollinearity check of covariates.**

|  | Step 1 |
| --- | --- |
| Body Temperature (0.5h) | 2.1 |
| Body Temperature (2h) | 2 |
| Lowest body temperature (within 24h) | 1.3 |
| Highest temperature | 1.4 |
| Sex | 1.3 |
| Age | 2.2 |
| Heart rate | 1.4 |
| Systolic pressure | 1.8 |
| Pulse oxygen saturation | 1.3 |
| Time from onset to visit | 1.4 |
| Heatstroke Type | 2.4 |
| Kidney failure | 1.5 |
| Diabetes | 1.3 |
| Stroke | 1.2 |
| Mental Disorder | 1.2 |
| Coronary heart disease | 1.3 |
| Hypertension | 1.5 |
| Ambient temperature | 1.2 |
| Glasgow Coma Scale | 1.8 |

**TABLE S2. Associations of covariates with hospital mortality.**

| Covariates | N | OR （β） | 95%CI Low | 95%CI Up | P.value |
| --- | --- | --- | --- | --- | --- |
| Sex | 143 | 2.69 | 0.83 | 8.74 | 0.100 |
| Age | 143 | 1.03 | 0.99 | 1.07 | 0.164 |
| Heart rate | 143 | 1.01 | 0.99 | 1.04 | 0.375 |
| Systolic pressure | 143 | 0.99 | 0.97 | 1.01 | 0.436 |
| Pulse oxygen saturation | 143 | 0.90 | 0.84 | 0.97 | 0.004 |
| Time from onset to visit | 143 | 0.92 | 0.76 | 1.11 | 0.371 |
| Heatstroke Type | 143 | 0.89 | 0.27 | 2.94 | 0.849 |
| Kidney failure | 143 | inf. | 0.00 | Inf | 0.990 |
| Diabetes | 143 | 5.12 | 0.86 | 30.60 | 0.073 |
| Stroke | 143 | 0.90 | 0.10 | 7.75 | 0.923 |
| Mental Disorder | 143 | 2.33 | 0.57 | 9.56 | 0.242 |
| Coronary heart disease | 143 | 1.31 | 0.15 | 11.54 | 0.808 |
| Hypertension | 143 | 1.15 | 0.29 | 4.56 | 0.838 |
| Ambient temperature | 143 | 1.04 | 0.85 | 1.28 | 0.687 |
| Glasgow Coma Scale | 143 | 0.65 | 0.48 | 0.87 | 0.005 |

**TABLE S3. Changes in the estimates of body temperature for hospital mortality in HS patients.**

|  | Body Temperature (0.5h) | | Body Temperature (2h) | | Lowest body temperature (within 24h) | |
| --- | --- | --- | --- | --- | --- | --- |
|  | Basic model | Complete model | Basic model | Complete model | Basic model | Complete model |
| Initial regression coefficient | 0.09 | 0.10 | 0.77 | 1.39 | -1.17 | -2.06 |
| Covariates |  |  |  |  |  |  |
| Sex | 0.12 * | 0.05 * | 0.84 | 1.13 * | -1.25 | -1.84 * |
| Age | 0.11 * | -0.06 * | 0.84 | 1.05 * | -1.23 | -1.94 |
| Heart rate | 0.09 | 0.01 * | 0.78 | 1.38 | -1.15 | -1.71 * |
| Systolic pressure | 0.07 * | 0.13 * | 0.75 | 1.26 | -1.15 | -2.05 |
| Pulse oxygen saturation | 0.03 * | 0.17 * | 0.76 | 1.43 | -1.01 * | -2.18 |
| Time from onset to visit | 0.12 * | 0.20 * | 0.79 | 1.41 | -1.16 | -2.03 |
| Heatstroke Type | 0.09 | 0.10 | 0.77 | 1.15 * | -1.21 | -1.95 |
| Kidney failure | 0.06 * | 0.09 * | 0.80 | 1.42 | -1.50 * | -1.86 |
| Diabetes | 0.01 * | 0.20 * | 0.71 | 1.42 | -1.50 * | -1.91 |
| Stroke | 0.09 | 0.09 | 0.77 | 1.40 | -1.17 | -1.92 |
| Mental Disorder | 0.11 * | -0.05 * | 0.78 | 1.12 * | -1.14 | -1.80 * |
| Coronary heart disease | 0.10 | 0.09 | 0.78 | 1.39 | -1.19 | -2.00 |
| Hypertension | 0.09 | 0.11 | 0.77 | 1.39 | -1.19 | -2.07 |
| Ambient temperature | 0.10 | 0.10 | 0.77 | 1.38 | -1.19 | -2.06 |
| Glasgow Coma Scale | 0.01 * | 0.02 * | 0.76 | 1.02 * | -0.93 * | -2.03 |

* Represents a change of more than 10% from the initial regression coefficient.

**TABLE S4. summary of the confounders sellected in the adjusted models**

| outcomes | Body Temperature | **confounders sellected** |
| --- | --- | --- |
| Hospital mortality | Body Temperature (0.5h) | initial temperature, sex, age, heart rate, systolic pressure, pulse oxygen saturation, Glasgow Coma Scale, time from onset to admission, kidney failure, diabetes, and mental disorders |
|  | Body Temperature (2h) | initial temperature, sex, age, pulse oxygen saturation, Glasgow coma scale, stroke type, diabetes, and mental disorders |
|  | Lowest body temperature (within 24h) | initial temperature, sex, heart rate, pulse oxygen saturation, Glasgow Coma Scale, kidney failure, diabetes, and mental disorders |
| Organs damaged | Body Temperature (0.5h) | initial temperature, heart rate, systolic pressure, pulse oxygen saturation, Glasgow Coma Scale, and time from onset to admission |
|  | Body Temperature (2h) | initial temperature, heart rate, systolic pressure, pulse oxygen saturation, Glasgow Coma Scale, and hypertension |
|  | Lowest body temperature (within 24h) | initial temperature, heart rate, systolic pressure, pulse oxygen saturation, Glasgow Coma Scale, and diabetes |
| Number of damaged organs | Body Temperature (0.5h) | initial temperature, heart rate, systolic pressure, pulse oxygen saturation, Glasgow Coma Scale, diabetes, and mental disorders |
|  | Body Temperature (2h) | initial temperature, heart rate, systolic pressure, pulse oxygen saturation, Glasgow Coma Scale, and mental disorders |
|  | Lowest body temperature (within 24h) | initial temperature, heart rate, systolic pressure, pulse oxygen saturation, Glasgow Coma Scale, and mental disorders |
| Neurologic sequelae at discharge | Body Temperature (0.5h) | initial temperature, age, heart rate, systolic pressure, pulse oxygen saturation, Glasgow Coma Scale, diabetes, stroke, mental disorders, coronary heart disease, and hypertension |
|  | Body Temperature (2h) | initial temperature, age, heart rate, systolic pressure, pulse oxygen saturation, Glasgow Coma Scale, diabetes, and mental disorders |
|  | Lowest body temperature (within 24h) | initial temperature, heart rate, systolic pressure, pulse oxygen saturation, Glasgow Coma Scale, kidney failure, diabetes, and mental disorders |

**TABLE S5. The segmented regression of body Temperature and the number of damaged organs**

| Body Temperature (0.5h) | | Lowest body temperature (within 24h) | |
| --- | --- | --- | --- |
| < 38.5℃ | -0.57 (-1.54–0.40) 0.254 | ≤ 36℃ | -1.25 (-2.01– -0.49) 0.002 |
| 38.5 - 40.0℃ | 0.27 (-0.31–0.86) 0.363 | > 36℃ | 0.02 (-0.47–0.51) 0.939 |
| > 40.0℃ | 1.42 (0.29–2.56) 0.015 |  |  |
| Log Likelihood Ratio Tests | 0.007 | Log Likelihood Ratio Tests | 0.016 |
